# Understanding hairy cell leukemia in the context of mature B−cell neoplasms: tumor microenvironment and extracellular vesicle contribution to disease pathogenesis

**DOI:** 10.3389/fimmu.2026.1741794

**Published:** 2026-02-18

**Authors:** Vincenzo Ingangi, Dominga Amoroso, Eugenia Passaro, Vincenzo Di Vaia, Filippo Maltoni, Ghazal Narimanfar, Michele Minopoli, Rita Rosa, Mariateresa Ametrano, Elena Di Gennaro, Alessandro Broccoli, Pier Luigi Zinzani, Lucia Catani, Chiara Ciardiello

**Affiliations:** 1 Preclinical Models of Tumor Progression Unit- Istituto Nazionale Tumori-IRCCS- Fondazione G. Pascale, Naples, Italy; 2 Experimental Pharmacology Unit, Istituto Nazionale Tumori –IRCCS– Fondazione G. Pascale, Naples, Italy; 3 IRCCS Azienda Ospedaliero-Universitaria di Bologna, Institute of Hematology “L. e A. Seràgnoli”, Bologna, Italy; 4 Department of Medical and Surgical Sciences, University of Bologna, Bologna, Italy

**Keywords:** 3D models, bone marrow fibrosis, extracellular vesicles, hairy cell leukemia, immuno evasion, mature B cell neoplasms, tumor microenvironment

## Abstract

Mature B-cell neoplasms constitute a biologically and clinically heterogeneous group of hematologic malignancies, defined by the clonal proliferation and accumulation of monotypic mature B lymphocytes, which may involve the peripheral blood, bone marrow (BM), lymphoid tissues, or present primarily in extranodal sites. While the pathogenesis of common subtypes, such as chronic lymphocytic leukemia, multiple myeloma, Hodgkin Lymphoma and Diffuse Large B-cell Lymphoma has been extensively studied, those of rare entities like Hairy Cell Leukemia (HCL) remains poorly understood. HCL is a distinct B-cell neoplasm marked by BM infiltration of atypical “hairy” cells, pancytopenia, BM fibrosis, and the BRAF V600E mutation, which is a defining molecular hallmark of the classic form of the disease. Analogous to solid tumors, growing evidence shows that the tumor microenvironment (TME) is a pivotal contributor in both the initiation and progression of all these malignancies. However, in rare mature B-cell neoplasms, understanding how tumor cells interact with their microenvironment, in terms of immune invasion, stromal crosstalk, and tissue remodeling, remains a challenge, partly due to the scarcity of patient samples and limited availability of preclinical models. In the context of TME, extracellular vesicles (EVs) have emerged as central mediators of intercellular communication within both solid and hematological malignancies. In line with numerous findings from solid tumors, EVs are receiving heightened attention as key mediators of disease progression, immune modulation, and treatment response in blood tumors, by modulating cellular interactions and delivering bioactive cargo in the tumor milieu. This review presents HCL within the broader spectrum of mature B-cell neoplasms, highlighting the current state of knowledge on the dynamic crosstalk between malignant B cells and their TME. Particular attention is given to EVs, which play key immuno-regulatory roles by interacting with both immune and non-immune components of the TME, including stromal cells. We explore how EVs contribute to disease pathogenesis, offering a unifying framework for integrating complex interactions that are often under-investigated in rare disease contexts. Building on this synthesis, we propose that the insights gained from well-characterized lymphoproliferative disorders may serve as a valuable foundation for investigating related yet poorly understood conditions, such as HCL. Furthermore, given the scarcity of both biological samples and reliable preclinical models for rare hematological malignancies, we highlight the strategic role of European biobanks in providing access to well-annotated clinical samples—an essential resource for fostering interdisciplinary collaboration and enabling advanced experimental modelling.

## 1 Epidemiological trends and classification of mature B-cell neoplasms

Mature B-cell neoplasms constitute a biologically and clinically heterogeneous group of hematologic malignancies, defined by the clonal proliferation and accumulation of monotypic mature B lymphocytes which may involve the peripheral blood, bone marrow (BM), lymphoid tissues or present primarily in extranodal sites. They encompass diverse entities differing in morphology, immunophenotype, clinical behavior, and genomic features. For epidemiologic purposes, they are usually grouped into Non-Hodgkin lymphomas (NHL), Hodgkin lymphoma (HL), chronic lymphocytic leukemia (CLL), and multiple myeloma (MM), although each category includes multiple distinct subtypes (1).

Lymphomas commonly arise in lymph nodes (which appear enlarged in most instances); however, they may also involve primarily extranodal sites—such as the gastrointestinal tract or central nervous system, often with secondary BM infiltration (2–4). Mature B-cell leukemias and plasma cell neoplasms involve the BM in almost all cases (with an exception represented by extraosseous plasmacytoma). CLL, indeed, is by definition characterized by circulating mature neoplastic B-lymphocytes, which in turn re-circulate between marrow and lymph nodes (where the initial neoplastic events take place), and again between marrow and peripheral blood (5). Certain mature B-cell neoplasms recognized in the WHO-HAEM5 classification—such as splenic marginal zone lymphoma and related entities—are characterized by predominant splenic involvement as a defining feature and often accompanied by marrow infiltration and usually lacking significant lymph node enlargement. Hairy cell leukemia (HCL), for example, displays infiltration of splenic red pulp, hepatic sinusoids and portal tracts together with a low circulating leukemic burden (6). Lastly, MM is characterized by skeletal lytic lesion, resulting from increased bone resorption due to dysregulated bone remodeling driven by the complex crosstalk among neoplastic plasma cells, BM stromal cells, osteoclasts, and osteoblasts (7).

Classification systems have evolved substantially (8–13). The REAL classification (1994) introduced a multidimensional diagnostic model incorporating morphology, immunophenotype, clinical data, and genetics. This framework was expanded by WHO classifications in 2001, 2008, and 2017 (11–13). Advances in genomics led to the 2021 International Consensus Classification and the 2022 WHO-HAEM5, reflecting updated molecular knowledge and clinical insights (14, 15). Clinical context—age, disease distribution, exposure history, organ involvement, and blood counts—remains essential for accurate diagnosis (15).

Epidemiological data (GLOBOCAN) show rising global incidence and mortality of B-cell malignancies, underscoring the need for continued research (14, 16, 17). Although rare entities such as HCL are not specifically reported, HCL accounts for ~2% of leukemias and ~1% of lymphoid neoplasms, with an incidence of 0.3/100,000 per year (18, 19). Classic HCL is an indolent but incurable B-cell malignancy affecting predominantly middle-aged to older men (20, 21). Its rarity underscores the need for tailored approaches within low-incidence subgroups.

## 2 From germinal centers to malignancy: the cellular and molecular basis of mature B-cell neoplasms

Clonal expansion of B-cells and antibody affinity maturation occur within germinal centers (GCs), which form in B-cell follicles of secondary lymphoid organs after antigen stimulation. Naïve B-cells receiving follicular helper T-cell (Tfh) support, migrate into follicles where they initiate GC formation (22).

Within the GC, B cells proliferate rapidly and undergo somatic hypermutation of immunoglobulin variable genes. Mutated clones are selected through interactions with follicular dendritic cells and Tfh cells, allowing only higher-affinity B-cell receptor variants to survive (23, 24). The WHO classification organizes mature B-cells neoplasms into 12 families (25) reflecting their GC or non-GC origin (15). Malignant transformation from a normal B-cell to a malignant clone is driven by a combination of chromosomal aberrations, somatic mutations, and epigenetic dysregulation, which disrupt physiological checkpoints of maturation and immune regulation.

CLL exemplifies this heterogeneity: mutated IgHV (M CLL) reflects post-GC memory B cell origin and indolent behavior (5, 16, 26–28), whereas unmutated IgHV (U CLL) derives from naïve pre-GC B cells and is associated with aggressive disease (29). Cytogenetic lesions—including del(13q14), del(11q22 23), trisomy 12, and del(17p12)—further guide prognosis and therapy, with trisomy 12 and del(17p12) being more frequent in U CLL than in post-GC M CLL (5, 30). Post-GC neoplasms include also HL (both classic HL and nodular lymphocyte-predominant HL), Diffuse large B-cell lymphomas (DLBCLs), Primary mediastinal large B-cell lymphoma (PMBCL), non-nodal Mantle cell lymphoma (nnMCL), MM and HCL. Gene-expression profiling identifies two DLBCL subtypes. Activated B-cell-like (ABC), a post-GC aggressive form driven by chronic active BCR signaling and NF-κB–activating mutations (*TNFAIP3, MYD88, CD79A/B, CARD11*), and Germinal Center B-cell–like (GCB), linked to lesions such as t (14, 18) and mutations in epigenetic regulators like *CREBBP* and *EZH2* (31). Indeed, GCB subtype cells present more specific mutations related to lymphomagenesis, such as t (14, 18)(q32;q21) translocation that activates BCL2 promoting survival, and mutations affecting epigenetic regulation (*CREBBP, EZH2*) (31). MCL subtyping shows nnMCL as a post-GC, indolent, IGHV-mutated, SOX11-negative entity, differentiating it from the conventional aggressive (cMCL) (32). A defining molecular feature of almost all classic HCL cases is the BRAFV600E mutation, found in virtually all patients with very few exceptions. This mutation plays a central role in disease pathogenesis by activating the RAF-MEK-ERK signaling pathway, which drives uncontrolled proliferation and enhances survival of the malignant hairy cells (33).

HCL is unique among all hematologic malignancies due to several peculiar morphologic, immunophenotypic, molecular, and clinical features with not only initial sensitivity to myelotoxic chemotherapy with purine analogs, but also frequent relapses accompanied by a progressive loss of response to these drugs with time, calling for new therapeutic strategies. The near-universal *BRAFV600E* mutation activates the RAF-MEK-ERK signaling, which, in turn, drives uncontrolled proliferation and enhances survival of the malignant hairy cells. AKT–mTOR alterations also hold significance as a prognostic marker in patients with HCL (33, 34). Although several new drugs (BRAF/MEK inhibitors, and anti-CD22 immunotoxin) have been explored, purine analogs remain the standard of care for these patients, mostly as frontline therapy. These agents induce durable and unmaintained complete response in more than 70% of patients, and the relapse rates are about 30% to 40% after 5 to 10 years of follow-up, with overall survival frequently longer than 20 years. The HCL-variant (HCL-V), recently renamed in the WHO-HAEM5 classification as “Splenic B-cell lymphoma/leukemia with prominent nucleoli” (15), differs biologically and is more treatment-resistant (35). Due to these peculiarities, this will not be discussed in the present review.

GC-derived neoplasms include Burkitt Lymphoma (BL), Follicular Lymphoma (FL), and GCB-DLBCL which frequently carry hallmark translocations—t (8, 14) in BL, t (11, 14) in MCL, and t (14, 18) in FL (36, 37). Additional copy number alterations (CNAs), single nucleotide variants (SNVs), and structural variants (SVs) involving *TP53, MYC, BCL2, EZH2*, and *KMT2D* further promote the malignant transformation (38). In light of this molecular heterogeneity, it is worth to highlight that advances in next generation sequencing (NGS), whole-exome sequencing (WES), RNA-seq, and circulating tumor DNA (ctDNA) analysis have transformed diagnosis and monitoring across B-cell malignancies (39).

Understanding cellular origin and molecular drivers remains essential for accurate classification and targeted management. The “cryptic” nature of HCL reflects its fully mature yet aberrant B-cell phenotype, which diverges from normal maturation pathways (40).

### 2.1 Immunophenotyping in mature B-cell tumor diagnostics

The diagnosis of B-cell neoplasms relies on integrating clinical features, morphology, molecular testing, and immunophenotyping, which together determine lineage and maturation stage (41). Multiple diagnostic techniques support this process (42). Immunohistochemistry (IHC) on lymph node or BM biopsies, provides diagnostic prognostic and predictive insights including eligibility for target therapies, such anti- Programmed Death-Ligand (PD-L)1, CD19, CD20 or CD30 therapies (43). Flow cytometry further refines classification by analyzing antigen expression at the single-cell level (44).

Mature malignant B-cells generally lack antigenic determinants characteristic of immature B-cell phenotypes, such as TdT and CD34, while expressing CD19, CD20, and CD22 (45). Distinctive immunophenotypes support differential diagnosis across entities (46–56).

HCL is characterized by clonal expansion of mature B-cells arrested at a late stage of differentiation, showing a strong light chain restricted immunoglobulin with bright expression of CD19, CD20, CD22 and CD200. Interestingly, HCs are usually negative for CD5, CD23, CD10, and CD27 and positive for CD11c, CD103, CD123, and CD25. Strong positivity for C-C Motif Chemokine Receptor (CCR)3, CCR6, C-X-C chemokine receptor type 4 (CXCR4), CD26, PD-1 and Annexin A1 is also observed. A strong dependence of hairy cells on extracellular stimuli has been described including interactions with BM stromal cells and matrix. However, the mechanisms governing these interactions are still unknown. Preliminary observations suggest that CD11c and CD103 promote stromal interactions and marrow retention (21, 48, 57, 58).

Adhesion molecules further influence disease behavior. In CLL, the integrins LFA-1(CD11a/CD18) and VLA-4 (CD49d/CD29)—especially in trisomy 12—enhance ICAM-1 and vascular cell adhesion molecule 1 (VCAM-1) binding and correlate with aggressiveness. In MM, LFA-1, VLA-4, CD44, and ICAM-1 mediate BM adhesion and drug resistance (59).

**Figure 1:**
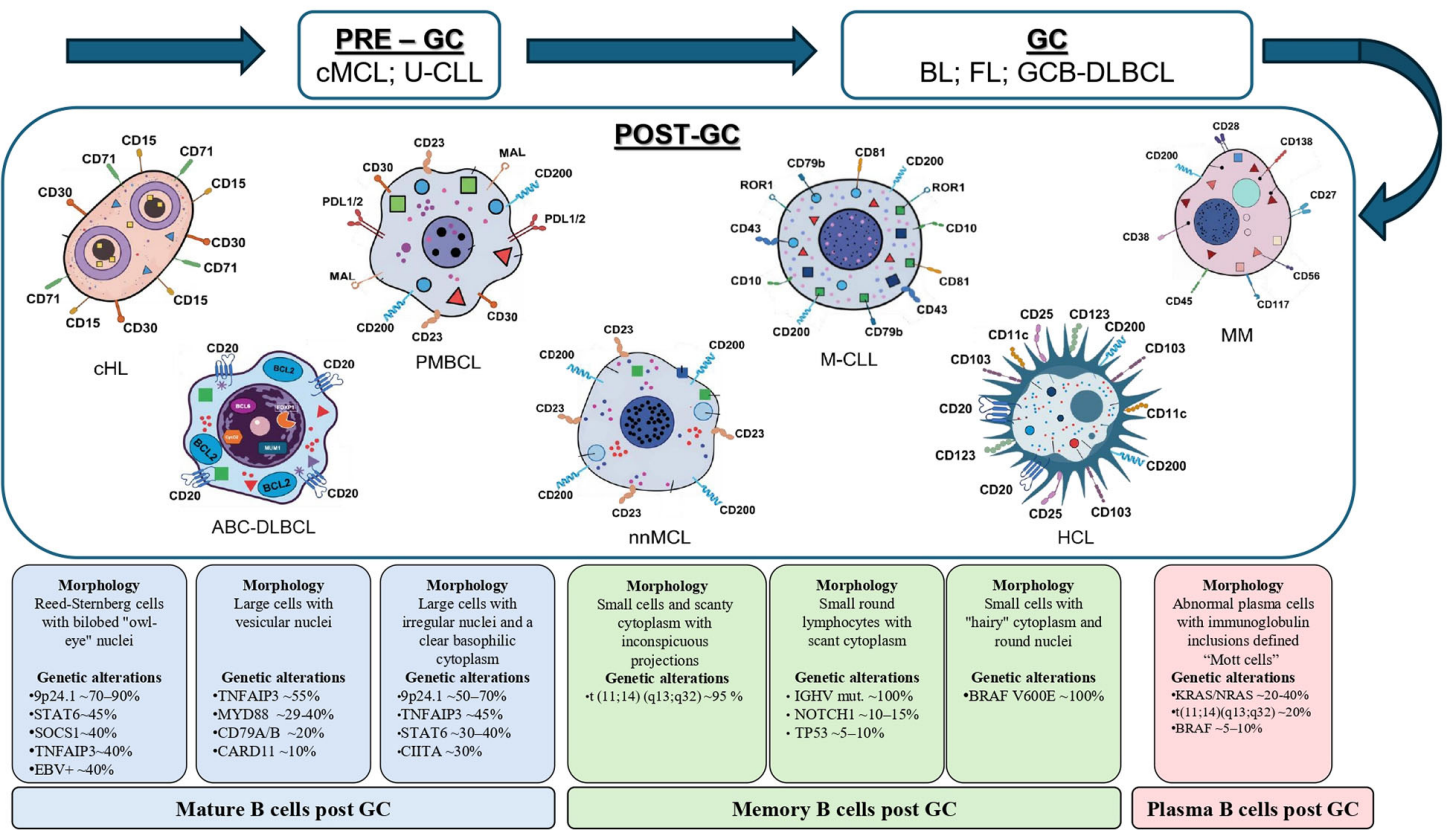
B-cell neoplasms: from ontogenesis to diagnosis. Schematic representation of 7 out of 12 families of Mature B Cell Neoplasms involving post–germinal center (post-GC) B lymphocytes, based on WHO 2022 classification. All seven shown entities are further organized according to their level of post-GC differentiation and displays main immunophenotype markers: Mature B cells evidenced in light blue: cHL (50), ABC-DLBCL (51), PMBCL (52); Memory B cells in light green: nnMCL (55), M-CLL (53, 54), HCL (48); Plasma B cells in light red: MM (47). Disease entities are also distinguished through a structured scheme combining morphology and genetic features (in boxes).

**Figure 1** resumes what we have pointed out on morphology, immunophenotype and genetic landscape of mature B-cell neoplasms.

### 2.2 Clinical features

Lymph node enlargement is the hallmark of lymphoproliferative disorders. Rapidly growing, asymmetric lymphadenopathy is typical of aggressive lymphomas such as DLBCL, BL and cMCL. HL characteristically presents supradiaphragmatic nodal disease, most frequently involving the mediastinum. The mediastinum represents the predominant or exclusive site of disease in PMBCL, often presenting as a bulky mass (>10 cm) with compression of thoracic structures. Indolent lymphomas such as FL and CLL usually show disseminated, symmetric lymphadenopathy with slow growth, although bulky abdominal or retroperitoneal masses may occur. BM involvement is frequent in indolent lymphomas but uncommon in aggressive subtypes; however, clinically significant BM failure with cytopenia occurs only in advanced disease and represents an indication for treatment. Extranodal involvement may be observed in all lymphoma subtypes. In DLBCL and BL it is usually synchronous with nodal disease and may cause organ dysfunction, whereas post-germinal center indolent lymphomas may even arise from the mucosa-associated lymphoid tissue of the gastrointestinal tract, the airways or the skin, without spreading to nodes, in these cases being defined as “primary extranodal”. Lymphocytosis is typical of CLL, though leukemic phases may occur in other NHLs, most commonly non-nodal MCL, especially in the most advanced phases. Splenomegaly is frequent in CLL and indolent lymphomas but rare in aggressive forms. MM is presented uniquely with BM plasma cell infiltration causing anemia, bone disease and hypercalcemia, and with a monoclonal protein leading to renal dysfunction or hyperviscosity.

Finally, HCL has a truly peculiar presentation: patients may be completely asymptomatic but showing at least one peripheral blood cytopenia (mainly thrombocytopenia or neutropenia found upon routine examinations), with a constant lack of circulating monocytes, and a variable degree of splenomegaly. Lymph nodes are generally not enlarged. Upon BM biopsy, always required to establish the diagnosis, hypoplasia is not an infrequent finding, along with an increase in reticulin fibrosis. Both these aspects may concur in causing cytopenias. Some patients, on the other hand, may be severely ill at onset due to a concomitant infection, which is favored by prolonged neutropenia and impaired immune response (57, 60–62). Rare extramedullary or immune-mediated manifestations have also been described (63).

## 3 The tumor microenvironment: a key player in hematologic malignancies vs. solid tumors

Over time, it has become clear that tumors are not isolated entities, but rather complex diseases involving genetic alterations, metabolic rewiring, persistent inflammation, and immune dysregulation. This understanding has shifted cancer research from a tumor-centric to a TME-centric paradigm, influencing every stage of patient care—from prevention to diagnosis and treatment (64, 65). The TME encompasses a dynamic network of stromal and immune cells, cytokines and chemokines (66), growth factors, extracellular vesicles (EVs) (67), and the extracellular matrix (ECM) (68), all of which interact with tumor cells to shape disease behavior.

In both solid and hematologic malignancies, the TME plays a critical role in promoting tumor progression and therapy resistance (69, 70). However, its structure and function differ markedly between these cancer types. In solid tumors, the TME is spatially confined around the tumor mass and includes cancer-associated fibroblasts, endothelial cells, pericytes, and infiltrating immune cells. Biomechanical signals such as hypoxia, pH gradients, and ECM remodeling influence not only tumor cell behavior but also immune and vascular responses (68, 71, 72). These dynamics contribute to T and Natural killer (NK) cell exhaustion, limiting their infiltration and cytotoxic activity (73). T cell exhaustion is also observed in mature B-cell neoplasms such as HL, DLBCL, and MM (74, 75).

In hematologic malignancies, the TME is more diffuse and systemic, primarily involving the BM, lymph nodes, and spleen. Unlike most organs where solid tumors arise, the BM is physiologically hypoxic, despite its rich vascularization (76, 77). This hypoxia varies across BM niches, with the endosteal niche being more hypoxic and enriched in hypoxia-inducible factor (HIF)-1α-positive cells compared to the vascular niche. Hypoxia plays a role in maintaining hematopoietic stem cells and becomes more pronounced in marrow-infiltrating malignancies like myeloma and lymphoma. In both solid and hematologic tumors, hypoxia triggers adaptive responses via HIFs, promoting angiogenesis, metabolic reprogramming, immune evasion, and resistance to therapy. Solid tumors often develop extensive hypoxic and acidic microenvironments due to rapid growth and poor perfusion, leading to anaerobic glycolysis and extracellular acidification. Hematologic malignancies, while typically lacking solid mass formation, can still generate localized hypoxic niches—especially in BM, but acidification of the tumor milieu is less pronounced (70, 78). Approximately 50–60% of solid tumors develop hypoxic regions (79), whereas hematologic tumors show greater heterogeneity. In leukemia, where malignant cells circulate widely, hypoxia has a limited impact and does not significantly influence endothelial progenitor cell behavior or angiogenesis (80, 81). Nonetheless, increased BM vascular density has been observed in leukemias and MM, suggesting that angiogenesis contributes to disease progression in hematologic cancers as well (82–84).

Metabolic dependencies also differ between tumor types. Leukemic cells are more isolated and highly reliant on specific metabolic pathways, making them particularly vulnerable to metabolic inhibition (85, 86). In contrast, metabolic targeting in solid tumors must be more selective to avoid collateral damage to surrounding non-tumor cells, including immune populations.

Therapeutic strategies increasingly aim to target immunological factors and synergistic changes in the TME to eradicate tumor cells (87). In solid tumors, this includes disrupting angiogenesis, remodeling the ECM, and reactivating immune responses via checkpoint blockade. In hematologic malignancies, interventions focus on disrupting stromal signaling, modulating cytokine networks, and overcoming immune dysfunction.

Exploring the similarities and differences between solid and hematologic tumors in processes such as T-cell depletion, angiogenesis, hypoxia, and metabolic adaptation is crucial for understanding tumor progression dynamics and for identifying targeted therapeutic strategies.

### 3.1 The role of the tumor microenvironment in mature B-cell neoplasms

As already pointed out, mature malignant B-cells exhibit a specific tropism for lymph nodes, BM, or other secondary lymphoid organs, where they find support of an advantageous niche for their survival and propagation (88–90).

Tumor B-cells themselves secrete chemoattractant chemokines (such as C-C motif chemokine ligand (CCL)3, CCL4) that recruit T cells and monocytes/macrophages and stimulate stromal components (91). Myeloid bystander cells, including tumor associated macrophages, often polarize towards an immunosuppressive phenotype under the influence of factors such as Interleukin (IL)-10, CSF-1, and Transforming Growth Factor (TGF)-β, further promoting tumor cell survival. Other soluble cytokines and growth factors in the TME include IL-4, IL-6, IL-8, Tumor Necrosis Factor (TNF)-α, and cell adhesion molecules such as VCAM-1, which actively contribute to malignant B cell progression (92–94).

In lymph nodes and secondary lymphoid organs, malignant B cells localize in proliferation centers where they engage in intensive cross talk with T cells (especially CD4^+^ helper T cells and Tfh cells), monocyte derived Nurse like cells, follicular dendritic cells, and stromal fibroblasts (28, 95, 96). Interestingly, in CLL a specific role has been described for Nurse like cells, which share features with leukemia-associated macrophages, able to act as an alternative source of CCL21 thereby sequestering malignant B-cells within lymph nodes (97). With a complex bidirectional crosstalk Nurse like cells strongly support CLL cells providing feed and protection from drugs but are also manipulated by leukemic cells in order to create an immunosuppressive milieu that allows immune evasion (95).

In the BM microenvironment, along with perivascular stromal cells, osteoblasts, endothelial cells and resident myeloid cells, a crucial role in tumor progression is played by mesenchymal stromal cells (MSCs) (98) that strongly cooperate with malignant mature B-cells conferring protection from apoptosis and fostering drug resistance with a mechanism involving Notch-1 signaling (99, 100).

B-cells neoplasms establish dynamic interactions with marrow stromal cells, transforming the BM TME into a tumor-supporting niche (101). Depending on the tumor subtype, different patterns of BM infiltration are observed: diffuse infiltration that is associated with an advanced stage and poor prognosis, nodular infiltration (e.g. CLL) that is considered less invasive with better prognosis and interstitial or perivascular infiltration (e.g. CLL, HCL, DLBCL) that represents an intermediate pattern in aggressiveness and clinical impact (86). To date, the mutational status of IgHV gene, distinguishing U CLL from M CLL, has been associated with different patterns of BM infiltration when coupled to ZAP-70 protein expression (102).

In addition to infiltrating the marrow, tumor cells actively modify the niche in an immunosuppressive environment that protects them and drives disease progression. These changes in the microenvironment may precede tumor-inducing mutations in hematopoietic cells, suggesting a non-cell-autonomous model of oncogenesis in which the environment itself contributes to tumor origin (103, 104). These observations reinforce the idea that the BM microenvironment is an active co-protagonist in hematological pathogenesis. For this reason, integrating knowledge of niches into clinical practice may represent a fundamental strategy to improve future treatments, making them more personalized and efficient (90).

Malignant B-cells home to the BM microenvironment through the interaction of their CXCR4 receptor with the stromal-derived factor-1 (CXCL12) and adhere through integrins, particularly with the α4β1 integrin (also known as very late antigen-4, VLA-4) binding to VCAM-1 expressed on mesenchymal and perivascular stromal cells (105). These VCAM-1/VLA-4 adhesion-mediated signals lead to chemoresistance through the activation of NF-κB (106).

In the specific case of MM, malignant plasma cells can affect the bone homeostasis dependent by deposition and resorption of osteoblasts and osteoclast respectively, leading to pathologic bone fractures and bone pain (107). Specifically, it is reported that MM cells are able to block the differentiation of mesenchymal stem cells into osteoblasts by producing both Wnt inhibitors or the epidermal growth factor (EGF) family member amphiregulin (108, 109). Moreover, in the MM bone microenvironment, elevated cytokines (such as TNF-α, interferon (IFN)-gamma, IL-1β, and IL-6) and direct contact with malignant plasma cells trigger osteoblast apoptosis, impairing new bone formation (110).

BM fibrosis and ECM composition play a crucial role in mature B-cell malignancies by supporting tumor survival, drug resistance, and microenvironmental remodeling. In particular, Tancred et al. analyzed the expression and localization of fibronectin, laminin, and collagens I and IV in the core biopsies of normal donors and patients with MM. They reported reduced levels of fibronectin and collagen I in MM patients with high-level plasmacytosis, whereas the expression of collagen IV in the BM of MM patients was higher than in the BM from normal donors (111). These findings have been subsequently confirmed by a proteomic characterization which highlighted the reduction and disorganization of type I collagen (COL1A1/COL1A2) and reported significant upregulation of ECM-associated proteins such as Annexin A2 and Galectin-1, which correlate with a poor prognosis in patients with MM (112). Conversely, in HCL, malignant hairy B-cells actively contribute to the abnormal ECM by synthesizing and assembling an insoluble fibronectin matrix increasing the characteristic BM fibrosis (113). Additionally, HCL is associated with elevated levels of TGF-β1, which stimulates fibroblasts to increase production and deposition of type I and type III procollagens, further altering ECM composition (114). The HCL cells bearing CXCR4 are strongly attracted within a CXCL12 rich microenvironment, highly expressed by stromal cells. HCL cells also secrete TNF-α, which upregulates VCAM-1 on endothelial and stromal cells, thereby enhancing their adhesion and migration. Stromal interactions in HCL can activate downstream signaling via integrin α4β1/VLA-4, triggering NF-κB pathways that sustain proliferation and reduce apoptosis (115). In HCL, the protective role of the microenvironment has been recently emphasized (115).

All these observations reinforce the idea that the tumor microenvironment is an active co-protagonist also in mature B-cell neoplasms. For this reason, integrating knowledge of niches into clinical practice may represent a fundamental strategy to improve future treatments.

### 3.2 Three-dimensional culture models in mature B-cell neoplasms

Three-dimensional (3D) cell culture models have become essential tools in cancer research, allowing researchers to investigate tumor growth, invasion, and response to therapies framed in a physiologically relevant context able to recapitulate the complex tissue architecture, oxygen gradients, and cell–cell interactions present *in vivo* (116). The 3D cell culture systems have traditionally been employed to investigate both primary solid tumors (117) and the metastatic process (118, 119). Emerging studies have also demonstrated the potential of 3D models in hematologic malignancies, where, by providing structural and spatial cues, they enable malignant cells to interact with stromal and endothelial counterparts within a more physiologically relevant context. This interaction promotes long-term survival and faithfully reproduces drug resistance mechanisms observed *in vivo* (120). These models have been successfully applied to both the acute and chronic form of hematological disease (121–123) and specifically in various mature B-cell neoplasms, as summarized in **Supplementary Table 1** (124–137). In this context, Barbaglio and collaborators developed a rotational 3D co-culture model using a porous gelatin-based scaffold (Spongostan) seeded with HS5 stromal cells and CLL cells (primary cells) or MEC1 cell line. The Rotating Cell Culture System ensured uniform nutrient diffusion and sustained 3D organization. This dynamic system was designed to functionally replicate the BM microenvironment, emphasizing the cellular cross-talk and retention mechanisms that underlie CLL persistence *in vivo*. The model effectively captured the cytoskeletal protein hematopoietic lineage cell-specific protein 1 (HS1)- and CXCR4-dependent adhesion, as well as ibrutinib-induced mobilization (124). Furthermore, the same 3D Spongostan-scaffold has been applied for the biophysical modeling of the lymphoid microenvironment, co-culturing stromal and endothelial cells under static and dynamic (by using microfluidic bioreactors) conditions. Unlike the rotational system, this perfusion-based platform enabled fine control of shear stress and fluid flow. In particular the introduction of flow enhanced tissue-like organization with cells aligned along flow trajectories, ECM components deposition (such as fibronectin, laminin, and collagen), and upregulation of mechanotransduction genes (e.g. *LMNA, FSCN1, PTK2, ICAM1, VCAM1*). Moreover, CLL cells (MEC1 cell line) circulated through the dynamically matured scaffolds displayed enhanced homing and adhesion, behaviors absent in static conditions (125).

Moving beyond conventional two-dimensional cultures toward 3D methods, there are the scaffold-free approaches applied by Ilic et al. in order to investigate the cross-talk between stromal and MM cells (126). Stromal cells spontaneously organized into compact spheroids characterized by reduced metabolic and proliferative activity, hallmarks of a quiescent stromal state. Although this model effectively captured certain aspects of stromal physiology and has also been efficiently applied for CAR-cytotoxicity testing (127), it fell short in reproducing functional MM MSCs interactions, as myeloma cells localized only to the spheroid periphery. The use of a collagen-based co-culture model allows to overcome these limitations, by embedding both MSCs and MM cells within a type I collagen matrix. This configuration supported a more homogeneous cellular organization, maintained stromal chemokine expression (such as CXCL12), and crucially recapitulated drug resistance dynamics, as MM cells displayed reduced sensitivity to bortezomib, consistent with cell adhesion–mediated drug resistance observed *in vivo* (126). While these models remain simplified representations of the BM niche, they also provide a compelling framework for advancing physiologically relevant 3D platforms in hematological research.

Expanding from myeloma to aggressive B-cell lymphomas, similar 3D approaches have been applied to DLBCL. Foxall et al. developed a physiologically relevant 3D culture system to model DLBCL and its interactions with the TME (128). They integrated fibroblasts (from adipose-derived stem cells), primary human monocyte-derived macrophages, and primary DLBCL tumor cells, all embedded within a type I collagen matrix to form multicellular spheroids. Notably, fibroblasts were functionally polarized into a lymphoid-like stromal phenotype via cytokine stimulation (IL-4, TNF-α, LT-α/β), leading to the expression of key lymphoid stromal markers such as podoplanin, ICAM-1, and VCAM-1. The resulting 3D architecture enabled spatially organized and sustained interactions between tumor, stromal, and immune cells, better approximating the structural complexity of the native lymphoma microenvironment. This system supported the survival of primary DLBCL cells and allowed for the functional investigation of the antibody-dependent cellular phagocytosis (ADCP) immune mechanism in response to rituximab (anti-CD20 mAb). Phagocytosis efficiency was shown to be sensitive to matrix density: higher collagen concentrations impaired ADCP, whereas lower matrix densities facilitated phagocytosis and significantly reduced DLBCL cell viability (128).

A different approach, still within the context of DLBCL, aimed at studying a physiologically relevant microenvironment, has been the use of a bone-derived 3D scaffold model (129). Using decellularized human trabecular bone preserving essential ECM components such as collagen type I, fibronectin, and laminin, the authors reconstructed a structurally and biochemically authentic scaffold capable of supporting stromal and tumor co-cultures. When repopulated with MSCs, the model effectively recreated features of the BM niche, allowing lymphoma–stroma interactions and test the drug-resistance: OCI-LY18 (germinal center B-cell-like) cells exhibited intrinsic resistance to doxorubicin, whereas NU-DUL-1 (activated B-cell-like) cells developed resistance only when co-cultured with MSCs, emphasizing the distinct yet complementary roles of ECM composition and stromal signaling in modulating chemoresistance (129). This study highlights how the native bone-derived ECM can act as both a structural and biochemical determinant of tumor behavior, offering a level of physiological relevance difficult to achieve with synthetic scaffolds. However, the static nature, the lack of vascular or immune components of this method still limits its ability to fully reproduce the complexity of the marrow milieu.

Despite their growing utility in biomedical research, 3D culture systems still face significant technical and translational challenges that hinder their widespread adoption. Reproducibility across laboratories remains a major concern, particularly given the heterogeneity of scaffold-based models, which often vary in physical properties such as stiffness and porosity, affecting experimental outcomes (138). Scaffold-free systems, while useful in certain contexts, are inherently less stable for non-adherent hematologic cells, limiting their applicability. Moreover, dynamic culture platforms, though capable of mimicking physiological conditions more closely, are technically complex and poorly suited for high-throughput workflows. Addressing these limitations will require concerted efforts toward standardization, integration of real-time monitoring technologies, and the development of hybrid platforms that combine biomimetic scaffolds with relevant stromal and immune components under perfused or mechanically active conditions (120).

Considering the relevance of EVs in the dynamics between tumor and TME, it is worth to mention that, to the best of our knowledge, there are currently no studies employing 3D cell culture models to investigate the effects of EVs in mature B-cell neoplasms, nor 3D models specifically developed for the study of HCL. However, it is reported, in a 3D bioreactor system, that the production of EVs from human adipose-derived MSCs resulted increased in concentration and purity compared to traditional 2D cultures, under both normoxic and hypoxic conditions (139). This highlights a critical need for the scientific community to advance toward the adoption of such 3D models in these specific contexts, as they better recapitulate relevant physiological aspects and may provide more accurate insights into disease mechanisms and therapeutic responses.

## 4 From “Platelet dust” to immune modulators: the evolving understanding of extracellular vesicles

In the late 1960’s, EVs were initially described as “platelet dusts”, cell debris or experimental artifacts (140), while nowadays their existence as lipid bilayer particles released by all cell types under both physiological and pathological conditions and detectable in both tissues (141, 142) and body fluids (143–145) is well established. The growing complexity and heterogeneity of EVs, due to their diverse origins, densities, sizes, and molecular cargos, brought to their classification into distinct populations to refine the comprehension on their biological roles (146). Indeed, according to biogenesis mechanisms, EVs can be classified into the following main categories: I) “exosomes”, secreted upon plasma membrane fusion with multivesicular bodies (MVBs) of endosomal origin (147); II) “microvesicles” and “ectosomes”, plasma membrane-derived microparticles, enriched in cytosolic and plasma membrane-associated proteins (148); III) apoptotic bodies released from cells undergoing programmed cell death or apoptosis and contain both cytosolic components and nuclear fragments (149); IV) the recently emerged population of non-vesicular extracellular nanoparticles, comprising exomeres and supermeres (150, 151).

To date, according to the latest guidelines released by the International Society for Extracellular Vesicles (ISEV) on the minimal information for studies on EVs (MISEV2023), EVs can be categorized based on their size, regardless of the biogenesis mechanism: small EVs (S-EVs), which have a size ≤200 nm, and large EVs (L-EVs) ≥ 200 nm (146).

Interestingly, one of the earliest reports documenting the release of membrane-bound vesicles by tumor cells dates back to 1978, when “rare, pleomorphic membrane-lined particles” were observed in cell lines derived from patients with Hodgkin’s disease (152). This initial observation was soon followed, in 1979, by an independent study that identified plasma-derived vesicles released by murine leukemia cells (153), further supporting the notion that vesicle shedding is a conserved feature of malignant hematopoietic cells. From these pioneering studies, tumor derived EVs have emerged as key messengers in the development of both solid (154) and hematological malignancies (155). The ability to transfer bioactive molecules such as proteins, lipids and nucleic acids (DNA, RNA, miRNA) between tumor cells themselves and microenvironment cells, promotes functions such as angiogenesis (156), tumor cell migration (157, 158) and immune modulation (159).In 1996, antigen-presenting ability of EVs was highlighted for the first time, demonstrating that exosomes derived from B lymphocytes carried both MHC class I and II molecules, allowing them to present tumor antigen peptides to CD8+ and CD4+ T cells, acting as immunostimulatory factors in T cell responses (159). Conversely, EVs have been reported to inhibit the immune system response by hindering immune surveillance and creating an immunosuppressive microenvironment (160, 161).

Altogether, evidence from hematologic tumor microenvironments suggests that EVs are not merely passive byproducts of cellular activity but active mediators of immune modulation, potentially shaping innate and adaptive immunity through the induction of T-cell exhaustion, recruitment of monocytes, and polarization of macrophages toward an M2-like phenotype—as we discuss in detail in the next section.

### 4.1 Extracellular vesicles: key mediators of immune evasion in mature B-cell malignancies

This overview highlights the central and multifaceted role of EVs as critical mediators of immune modulation and tumor progression across various mature B-cell malignancies, including Lymphoma cHL, DLBCL, MM, CLL.

In cHL, Reed/Sternberg (HRS) cells release CD30+ EVs that stimulate IL-8 release from distant immune cells expressing CD30 ligand (162). IL-8, in turn, can induce immunosuppression by promoting the trafficking of neutrophils and myeloid-derived suppressor cells into the TME (163). Beyond these paracrine effects on innate immune cells, HRS cells engage in bidirectional communication with lymph node MSCs via EVs expressing ADAM10, one of the most prominent members of the Surface-expressed proteases of the disintegrin and metalloproteinase (ADAM) family. In particular, HRS cell-derived EVs induce MSCs to release MHC class I polypeptide–related sequences, whereas MSC-derived EVs stimulate TNF-α and CD30 production by HRS cells, collectively subverting host immune responses (164). In DLBCL, EVs derived from lymphoma B-cells and harboring mutated MYD88 activate proinflammatory signaling in mast cells and macrophages, reshaping the BM microenvironment into a pro-tumorigenic niche (165): however, although MYD88 mutations occur in ~40% of DLBCL, their attribution to DLBCL-derived EVs should be interpreted cautiously, as this mutation is nearly universal in Waldenström’s macroglobulinemia, which evolves into DLBCL in only a small fraction (~10%) of cases (166). Also, DLBCL-EVs promote macrophage M2 polarization via increased PGC-1β expression, facilitating tumor progression (167). EVs containing neuron-specific enolase (NSE) induce M2 polarization by disrupting the NF-κB pathway and enhancing macrophage migration (168). In another report, exosomal NSE have been reported to enhance glycolysis through the GSK3β/β-catenin/c-Myc pathway, driving M2-like macrophage differentiation (169).

EV-associated miRNAs also modulate innate immunity; in particular, acting on both macrophages and NKs functions. Indeed, reduced miR-7e-5p expression in DLBCL cells limits its transfer through EVs, leading to increased Fas Ligand (FASL) expression and activation of apoptotic caspase signaling in M1 macrophages (170); while plasma-derived EVs from DLBCL patients have been reported to suppress NK cell proliferation and cytotoxicity (171). By contrast, DLBCL EVs derived by *in vitro* cell lines have been reported as able to stimulate dendritic cell-mediated antitumor immunity, inducing T cell clonal expansion and IL-6 and TNF-α secretion, while decreasing the production of IL-4 and IL-10 immunosuppressive cytokines (172), highlighting context-dependent immune effects.

Despite these mechanistic insights, clinical translation remains uneven. Prognostic associations with proteins/miRNAs (173) illustrate the potential of EVs as biomarkers, yet methodological variability and limited integration with patient phenotypes persist. Standardized EV isolation, multi-omic profiling, and careful disease stratification will be essential to translate EV biology into actionable biomarkers and therapeutic targets in B-cell malignancies.

In MM, EVs have emerged as orchestrators of immune suppression, angiogenesis, and BM niche remodeling (174, 175). Regarding the cellular components of the innate immune response, NK cells showed reciprocal tumor-supportive interactions with myeloma cells: under genotoxic stress, MM cells release Heat Shock Protein (HSP)70^+^ EVs that activate NK cells via TLR2/NF-κB, increasing IFN-γ secretion without improving cytotoxicity (176). Conversely, other MM-EVs downregulate activating receptors NKG2D and NKp46, impairing NK-mediated killing (177). Notably, NK-derived EVs containing perforin and granzymes selectively induce apoptosis in drug-resistant MM cells, sparing normal hematopoietic cells (178).

MM-EVs also influence myeloid cells. Under hypoxia, EVs enriched in miR-1305 drive macrophage polarization toward an M2-like phenotype that promotes immunosuppression (179). Among the intercellular communication mechanisms emerged in the cross-talk with adaptive counterparts of the immune system, it has been observed that MM-EVs suppress T-cell activation and effector function through checkpoint molecules such as PD-L1, PD-1, and HLA-G, driving T-cell exhaustion (180). Additionally, EV-associated factors, including IL-8, SLC1A5, and sphingosine kinase-1 (SPHK1), further enhance immunoregulatory signaling (181). MM-EVs also disrupt the balance among CD4^+^, CD8^+^, and regulatory T-cell subsets, reducing cytotoxicity while increasing Treg-mediated suppression (182). Moreover, MM-EVs expressing CD39, CD73, CD38, and CD203a catalyze adenosine production, inhibiting T-cell activity and enhancing M2 polarization (183). MM-EVs interfere with dendritic cell differentiation via TGF-β1 signaling (174) and expand myeloid-derived suppressor cells through STAT3/STAT1 activation (184). Conversely, immune-cell-derived EVs—such as B-cell EVs carrying miR-335—are able to suppress MM by downregulating SOX4 and inhibiting the PI3K/AKT/HIF-1α pathway (185).

Consistent with observations in other hematologic malignancies, CLL-EVs have likewise emerged as pivotal regulators of leukemic biology and immune modulation. Plasma or serum EVs levels are elevated in CLL patients compared with healthy controls (186–190). Interestingly, CLL-EVs promote immune evasion primarily by impairing T-cell function through PD-L1 and TGF-β, suppressing activation and proliferation. They foster the expansion of regulatory T-cells and myeloid-derived suppressor cells while inducing T-cell exhaustion (191, 192). CLL-EVs disrupt T-cell receptor signaling (193) and enhance CD4^+^ T-cell motility and interaction with tumor cells (194). Functionally, these EVs suppress CD8^+^ T-cell cytotoxicity, facilitating leukemia expansion in murine models, where elevated EV-related gene expression correlates with poor survival (195).

In parallel, CLL-EVs downregulate NK cell receptors such as NKG2D, reducing cytotoxic activity (196). They also transfer oncogenic miRNAs, including miR-150 and miR-155, that enhance leukemic B-cell survival (191) and alter normal B-cell function. Exosomal RNA cargo further drives metabolic reprogramming, cell proliferation, and PI3K-mTOR pathway activation (197). Moreover, CLL-EVs exert profound effects on myeloid cells. They reprogram monocytes toward PD-L1^+^ and IL-10–secreting macrophages (191), while M2 macrophage-derived EVs in turn enhance CLL survival (198). Moreover, CLL EVs promote the differentiation of nurse-like cells via RNA-dependent mechanisms (199). Conversely, EVs from NK and cytotoxic T-cells deliver cytotoxic proteins with anti-leukemic effects (200).

In summary, EVs have emerged as central players of immune modulation across mature B-cell malignancies. EV-mediated crosstalk within the TME is highly dynamic and bidirectional, encompassing intricate interactions between malignant cells and various immune subsets through the transfer of bioactive proteins, lipids, and nucleic acids. This leads to immune evasion, tumor growth, and therapy resistance. Despite their shared abilities, the impact of EV-mediated signaling varies among diseases. In cHL and DLBCL, EVs remodel immune and stromal compartments, mainly promoting macrophage polarization and cytokine dysregulation. In MM, they reinforce immune suppression by impairing T and NK cell cytotoxicity while supporting the pro-tumor myeloid niche. Similarly, in CLL, they reprogram the immune response and propagate oncogenic signaling through RNA transfer. Altogether, these findings highlight EVs as pivotal mediators in the interplay between cancer and the immune system, acting both as facilitators of immune escape and as emerging targets for therapeutic intervention—thus opening new avenues in precision oncology.

**Figure 2:**
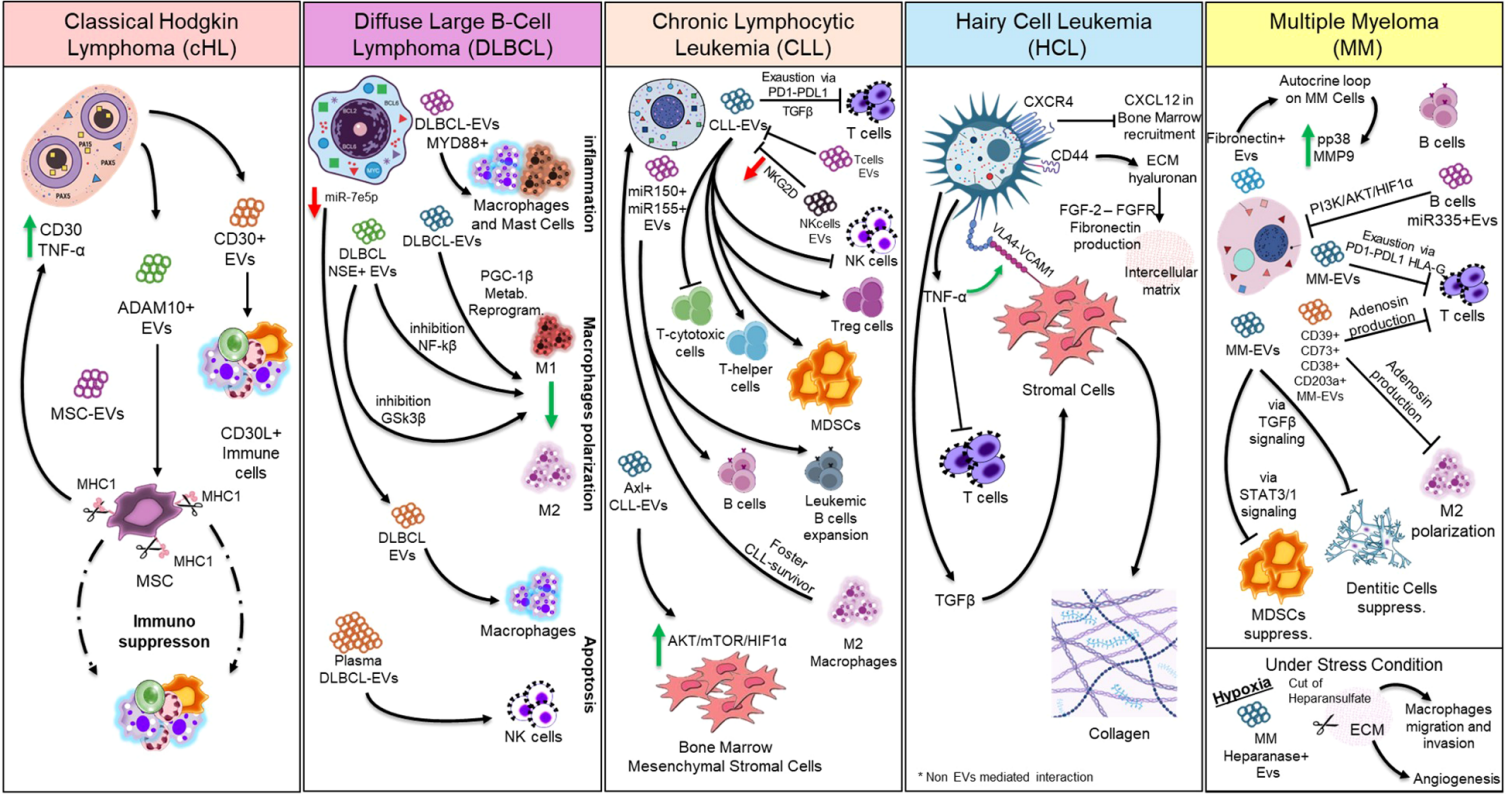
Distinctive molecular crosstalk between malignant B-cells and TME. The figure illustrates the principal EV-mediated crosstalk dynamics between malignant mature B-cells and the TME in cHL, DLBCL, CLL, and MM. In HCL, tumor–TME interactions are depicted as non-EV-mediated due to the current lack of supporting studies.

Although no published data currently exist on EVs in HCL, the disease is characterized by BM fibrosis and profound cytopenias, which are clinically associated with a markedly increased susceptibility to infections. This underscores that immune cells are not only locally affected—through stromal remodeling and the migration and expansion of HCL cells within the marrow—but are also systemically compromised. The immunological dysregulation in HCL represents a central axis of disease progression, with systemic immunosuppression exposing patients to recurrent and potentially life-threatening infections. On this basis, a multicenter clinical trial (NCT06774677) is underway to investigate how HCL-derived EVs modulate key components of the TME, including immune and stromal elements, with the aim of elucidating their role in both local niche conditioning and systemic immune impairment. **Figure 2** resumes what we have pointed out in the present section.

### 4.2 Extracellular vesicles and stromal–ECM remodeling: implications for bone marrow fibrosis in HCL

To mediate cell-to-cell communication, EVs must traverse the ECM from their cell of origin to recipient cells. Recent studies on ECM stress relaxation have shown that the mechanical properties of both the ECM and EVs enable their diffusion through the extracellular space, despite EVs having a larger diameter than the ECM mesh width (201).

In solid malignancies, EVs have been extensively reported to carry matrix metalloproteinases (MMPs), which play a central role in ECM remodeling. These enzymes not only degrade ECM components (141, 202) but also modulate signaling molecules within the extracellular space (203). In MM, exosomes released by hypoxic cells have been shown to contain elevated levels of heparanase, a proteolytic enzyme that cleaves heparan sulfate—an ECM-integrated glycosaminoglycan or cell surface component (204, 205). This activity promotes macrophage migration, invasion, and endothelial tube formation (206). Additionally, MM-derived EVs are enriched in surface fibronectin, which facilitates binding to heparan sulfate on target cells and mediates exosome–cell interactions (207). In cHL, EVs have been shown to express ADAM10, an enzyme responsible for ectodomain shedding. These ADAM10-active EVs can remotely influence recipient cells by serving as platforms for shedding processes, thereby extending the functional reach of cHL cells and shaping the immune response and tumor microenvironment beyond the primary site (164). E. Dumontet and colleagues have reviewed how the TME adapts in mature B-cell neoplasms, particularly within BM and lymph node niches, emphasizing the role of EVs in these process (89). In CLL, microvesicles detected in peripheral blood deliver the receptor tyrosine kinase Axl to BM stromal cells, activating the AKT/mTOR/p70S6K/HIF-1α axis and increasing VEGF synthesis (189). However, no studies have yet investigated the role of EVs in establishing a BM stromal niche in DLBCL. Although the involvement of EVs in HCL remains unexplored, a clinical trial (NCT06774677) is currently investigating their potential role in ECM–stromal remodeling, particularly in BM fibrosis. Central pathways driving fibrosis in HCL have been recently summarized (208). HCL cells bind hyaluronan—another ECM glycosaminoglycan—via CD44, leading to the release of FGF-2 into the intercellular matrix (209). This autocrine loop stimulates FGFR1 signaling and promotes fibronectin production (210, 211). Additionally, BM fibrosis is sustained by TGF-β–activated fibroblasts that produce collagen. **Figure 2** resumes what we have pointed out in the present section.

Recent findings in cardiac fibrosis offer further insight: activated BM cells release EVs enriched in miR-21a-5p, which upregulates Integrin αV (ITGAV) and enhances the synthesis of ECM proteins, particularly collagen type I alpha (Col1α), contributing to fibrotic remodeling (212).

These converging lines of evidence support the hypothesis that EVs may act as key modulators of ECM remodeling and fibrosis in HCL, warranting further investigation into their mechanistic roles and therapeutic potential.

## 5 Extracellular vesicles in hematology: infrastructure, methodology, and the unmet need in HCL

In hematological malignancies, national initiatives enable access to biological samples and clinical data. Disease-specific infrastructures for mature B-cell neoplasms include the UK CLL Biobank (UK), FILOThèque (France, CLL/AML), Blood Cancer Network Ireland (BCNI), and HEMLYM Biobank (Netherlands). The HARMONY Alliance integrates clinical and molecular data for AML, ALL, CML, and MM, creating a large-scale big data platform. Similarly, ENROL (EuroBloodNet) promotes registry harmonization for rare hematologic diseases. However, no dedicated biobank exists for HCL, revealing a major research gap. Blood Cancer United (LLS) supports research and patient services across blood cancers, including HCL. The HCL Foundation (USA) uniquely focuses on HCL, funding research and patient education. Biobanks enable standardized sample use, advancing rare disease research and precision medicine. Transparent procedures remain essential for reproducibility and reliability in translational research (213–215).

Within this framework, collection, storage and characterization of EVs – together with the definition of universal biomarker of EV characterization - remain critical methodological gaps that need to be addressed (213, 216). Both pre-analytical and analytical parameters critically affect the quantitative and qualitative evaluation of EVs. Pre-analytical variables such as blood collection procedures, sample processing, and storage conditions significantly influence EV yield, size distribution, and molecular composition (216, 217). Analytical variability—including isolation strategy, quantification method and marker selection —further contributes to inter-laboratory discrepancies (146). The harmonization of pre-analytical procedures, as promoted by national initiatives such as the GEIVEX strategy (Grupo Español de Investigación en Vesiculas Extracelulares) (216), represents an example of efforts to identify pre-analytical barriers through a web-survey, and promote a standardized workflows to reduce inter-laboratory variability. In addition, systematic collection of pre-analytical metadata is critical to identify hidden biases that may influence the characterization and analysis of EVs from biofluids. In this direction, the MIBlood-EV initiative (217). Minimal Information for Blood EV research, recently launched by the ISEV Blood EV Task Force, provides a reporting framework designed to: improve the quality of biobanking, promote the exchange between biobanks and laboratories of samples, facilitate inter-laboratory comparability of EV studies (218). Similarly, even analytical variability remains a major issue, as significantly affect the measurable fraction of circulating EVs (219). To address these challenges, the recently updated MISEV 2023 guidelines provide comprehensive recommendations for EVs isolation, characterization and functional studies reinforcing methodological rigor and reproducibility throughout all experimental stages of EVs research (146). In Italy, national initiative INNOVA – Italian network of excellence for advanced diagnostics (PNC- E3-2022–23683266 DIAGNOSTICA AVANZATA (HLS-DA) (https://www.innovaplatform.it/) - a Hub & Spoke network funded by the Italian Ministry of Health, aims to develop and disseminate standardized and advanced diagnostic methodologies across the country, integrating wet laboratory and bioimaging technologies that supports multistakeholder collaborations at both national and international levels. Taken together, these observations highlight that, although EVs are intrinsically stable structures, their quantitative and qualitative assessment is highly dependent on both pre-analytical and analytical factors. Therefore, establishing standardized and reproducible protocols for EVs is essential to ensure the reliability of their use as biomarkers or therapeutic agents (220). It follows that clinical trials can more reliably investigate the diagnostic, prognostic and therapeutic potential of EVs. The integration of clinical and biological data within clinical trials is essential to support target discovery, biomarker identification and patient stratification, fostering translational and collaborative projects (213). Although most ongoing clinical trials involving EVs are focused on solid tumors, their diagnostic and therapeutic potential in B-cell malignancies is increasingly acknowledged (221). A recent systematic review of EVs in clinical trials reported that, among them, nearly half (47.3%) of registered studies aimed at diagnostic, 33.3% of trials investigated companion diagnostics, the remaining 19.3% explored therapeutics applications (222). Methodological heterogeneity remains a key limitation (222). In this evolving landscape, the ongoing Italian multicenter, experimental, cross-sectional, non-profit clinical trial “Extracellular Vesicles-driven Communication in the Microenvironment of Hairy Cell Leukemia to Improve Patient Care Management” (NCT06774677), represents a significant step forward. It aims to elucidate how EVs derived from HCL cells contribute to immune dysfunction and BM fibrosis, potentially identifying an EV-based molecular signature associated with outcomes. As a result, integrating these rigorous practices will create a favorable landscape for translating preclinical discoveries into clinical application, providing new perspectives for hematological rare diseases in personalized medicine.

## 6 Conclusion

In summary, mature B-cell malignancies recapitulate many fundamental features of solid tumor microenvironments—including immune suppression, cytokine-mediated stromal remodeling, metabolic reprogramming, and hypoxia-driven adaptation—while maintaining distinctive hallmarks imposed by the organized architecture of lymphoid tissues and BM niches. Through the exploitation of homing and adhesion pathways, particularly the CXCR4–CXCL12 and VLA-4/VCAM-1 axes, malignant B cells establish reciprocal interactions with stromal and immune components that promote survival, drug resistance, and immune evasion (105). Unlike solid tumors, where the microenvironment is spatially confined, B-cell neoplasms orchestrate dynamic and systemic remodeling of multiple tissue niches, resulting in a diffuse yet functionally integrated tumor ecosystem (101). Comparative analysis of solid and hematologic TMEs may therefore uncover shared vulnerabilities and guide the development of therapeutic strategies aimed at disrupting protective niches and restoring effective anti-tumor immunity (64). Beyond the broad comparison between solid and hematologic TMEs, it is important to recognize that even within the BM, distinct homing niches coexist and evolve throughout disease progression. In MM, malignant plasma cells primarily colonize perivascular and endosteal regions, where interactions with osteoblasts, endothelial cells, and mesenchymal stromal cells foster immune evasion and bone destruction (107). In contrast, HCL cells preferentially reside in reticular and fibrotic areas enriched in CXCL12 and VCAM-1, which sustain their adhesion and survival through VLA-4– and NF-κB–dependent signaling. DLBCL exhibits a more heterogeneous marrow tropism, with interstitial or perivascular infiltration patterns that correlate with disease aggressiveness and clinical stage. These examples highlight that the BM cannot be regarded as a uniform sanctuary, but rather as a mosaic of specialized microanatomical niches whose composition, vascularization, and cellular cross talk are profoundly shaped by tumor subtype and progression (86). Understanding this intra-marrow heterogeneity will be essential to design therapeutic strategies capable of targeting the spatial and temporal diversity of tumor-supportive niches in B-cell malignancies (90). Finally, growing evidence highlights the multifaceted role of EVs in orchestrating ECM remodeling across hematologic malignancies (223) suggesting their potential involvement in the fibrotic processes characteristic of HCL. By integrating mechanical adaptability with bioactive cargo delivery, EVs may contribute to both local stromal reprogramming and systemic disease progression. Elucidating their function in HCL could not only deepen our understanding of marrow fibrosis but also unveil novel therapeutic targets for intervention.

## Glossary

ABC: Activated B-cell-like (DLBCL subtype)
ALL: Acute Lymphoblastic Leukemia
AML: Acute Myeloid Leukemia
BCL2: B-cell Lymphoma 2 (anti-apoptotic protein)
BCR: B-Cell Receptor
BL: Burkitt Lymphoma
BM: Bone Marrow
CARD11: Caspase Recruitment Domain-containing Protein 11
CAF(s): Cancer-Associated Fibroblast(s)
cMCL: Conventional Mantle Cell Lymphoma
CNA(s): Copy Number Alteration(s)
CLL: Chronic Lymphocytic Leukemia
CLL-EVs: Extracellular Vesicles derived from Chronic Lymphocytic Leukemia cells
CML: Chronic Myeloid Leukemia
CREBBP: CREB Binding Protein
ctDNA: Circulating Tumor DNA
CXCL/CXCR: C-X-C Motif Chemokine Ligand/Receptor
DLBCL: Diffuse Large B-Cell Lymphoma
DNA: Deoxyribonucleic Acid
ECM: Extracellular Matrix
ERK: Extracellular Signal-Regulated Kinase
EV(s): Extracellular Vesicle(s)
EZH2: Enhancer of Zeste Homolog 2
FISH: Fluorescence *In Situ* Hybridization
FL: Follicular Lymphoma
GC: Germinal Center
GCB: Germinal Center B-cell-like (DLBCL subtype)
GLOBOCAN: Global Cancer Observatory
HCL: Hairy Cell Leukemia
HCL-V: Hairy Cell Leukemia – Variant
HIF(s): Hypoxia-Inducible Factor(s)
HL: Hodgkin Lymphoma
HRS: Hodgkin and Reed–Sternberg Cells
ICC: International Consensus Classification
IgHV: Immunoglobulin Heavy-Chain Variable Region
IL: Interleukin
KMT2D: Lysine Methyltransferase 2D
M-CLL: Mutated Immunoglobulin Heavy Chain Variable Region Chronic Lymphocytic Leukemia
MCL: Mantle Cell Lymphoma
MM: Multiple Myeloma
MMP(s): Matrix Metalloproteinase(s)
MSC(s): Mesenchymal Stromal Cell(s)
MVB(s): Multivesicular Body(ies)
MYC: Myelocytomatosis Viral Oncogene
MYD88: Myeloid Differentiation Primary Response 88
NF-κB: Nuclear Factor Kappa-Light-Chain-Enhancer of Activated B Cells
NHL: Non-Hodgkin Lymphoma
nnMCL: Non-Nodal Mantle Cell Lymphoma
NGS: Next-Generation Sequencing
PMBCL: Primary Mediastinal Large B-Cell Lymphoma
REAL: Revised European-American Classification of Lymphoid Neoplasms
RNA-seq: RNA Sequencing
SNV(s): Single Nucleotide Variant(s)
SOX11: SRY-Box Transcription Factor 11
SV(s): Structural Variant(s)
TAM(s): Tumor-Associated Macrophage(s)
Tfh: Follicular Helper T Cell
TGF-β: T Transforming Growth Factor Beta
TNFAIP3: Tumor Necrosis Factor Alpha-Induced Protein 3
TNF-α: Tumor Necrosis Factor Alpha
TP53: Tumor Protein p53
TME: Tumor Microenvironment
U-CLL: Unmutated Immunoglobulin Heavy-Chain Variable Region Chronic Lymphocytic Leukemia
VEGF: Vascular Endothelial Growth Factor
WES: Whole-Exome Sequencing
WHO: World Health Organization
WHO-HAEM5: Fifth Edition of the WHO Classification of Hematolymphoid Tumors
